# Recreational Screen Use and Internalizing Problems from Preadolescence to Young Adulthood: A population-based Cohort and Co-Twin Control Study

**DOI:** 10.21203/rs.3.rs-7278950/v1

**Published:** 2025-08-22

**Authors:** Tong Gong, Emma Frans, Anna Ohlis, Shuyang Yao, Ruyue Zhang, Anders Nilsson, Yasmina Molero, Miriam Mosing, Isabell Brikell, Henrik Larsson, Paul Lichtenstein, Lisa Thorell, Patrik Magnusson, Ralf Kuja-Halkola, Yi Lu

**Affiliations:** Karolinska Insitutet; Karolinska Institutet; Karolinska Insitutet; Karolinska Institute; Karolinska institutet; Karolinska Insitutet; Karolinska Insitutet; Max Planck Institute for Empirical Aesthetics; Karolinska Institutet; Örebro University; Karolinska Institute; Karolinska Institutet; Karolinska Institutet; Karolinska Institutet; Karolinska Institutet

## Abstract

The mental health impact of increasing recreational screen use among youth has raised substantial concerns, yet questions about causality remain unresolved. Using data from ~22,000 Swedish twins followed from age 9 to 24, we examined associations between screen use and internalizing problems using multiple designs, including co-twin control comparisons, to strengthen causal inference. Associations between longer screen time and elevated internalizing symptoms during adolescence persisted in co-twin comparisons, supporting a potential causal link. Bidirectional associations were observed, with internalizing problems at younger ages also associated with later screen time increase. Adolescents who exceeded international screen time recommendations at ages 15 and 18 showed elevated internalizing symptoms at later ages, whereas those who reduced screen time to recommended levels did not. Heavy screen use at age 15, particularly during the weekdays, was associated with higher risk of clinical depression or anxiety. Findings support public health recommendations to limit recreational screen time.

## Introduction

The rapid proliferation of digital technologies has profoundly reshaped daily life, particularly for youth. The sharp rise in recreational screen use (*i.e*., screen-based entertainment via devices such as TV, computer or mobile phone^1^) has raised concerns about its potential impact on youth health, especially mental health^2^. Public health agencies worldwide have responded by introducing guidelines aimed at limiting recreational screen time among children and adolescents to prevent negative health effects^3–7^. However, these guidelines are primarily based on non-causal evidence; their effectiveness is thus under debate^8–10^. Rigorous investigations into causality and direction of effect are urgently needed to inform interventions on screen use among youth^10^.

Specifically for internalizing problems such as depression and anxiety, recent systematic reviews and meta-analyses of observational studies have generally supported an association with increased recreational screen use^11–13^, particularly among teenage girls^14,15^. Yet, observational studies inherently face limitations related to confounding, *i.e*., a wide range of developmental, environmental, social and psychosocial factors may simultaneously influence recreational screen use and internalizing problems, potentially biasing these observed associations^16^. Experimental designs such as randomized controlled trials (RCTs)—widely regarded as the gold standard for establishing causality—have begun to provide evidence supporting a causal link between recreational screen use and internalizing symptoms^17–19^. Nonetheless, practical constraints, for example restricting screen use in daily life (especially over extended periods), significantly limit the feasibility of conducting large-scale RCTs to explore cumulative exposure or long-term effects.

As a quasi-experimental design, twin research has long been used to strengthen causal inference^20^. Monozygotic (MZ) twins share their rearing environment and all inherited genetic factors, while dizygotic (DZ) twins also share their rearing environment and only (on average) half of their segregating genes. The co-twin control (or discordant twin) design, in which exposed twins are compared to their unexposed twin as matched controls, enables effective control of shared genetic and familial environmental factors typically unmeasured in observational studies^21^. Therefore, twin data offers a unique opportunity for investigating the potential causal link between screen use and internalizing problems, complementing RCTs findings with larger samples and longer follow-up.

**Figure 1:**
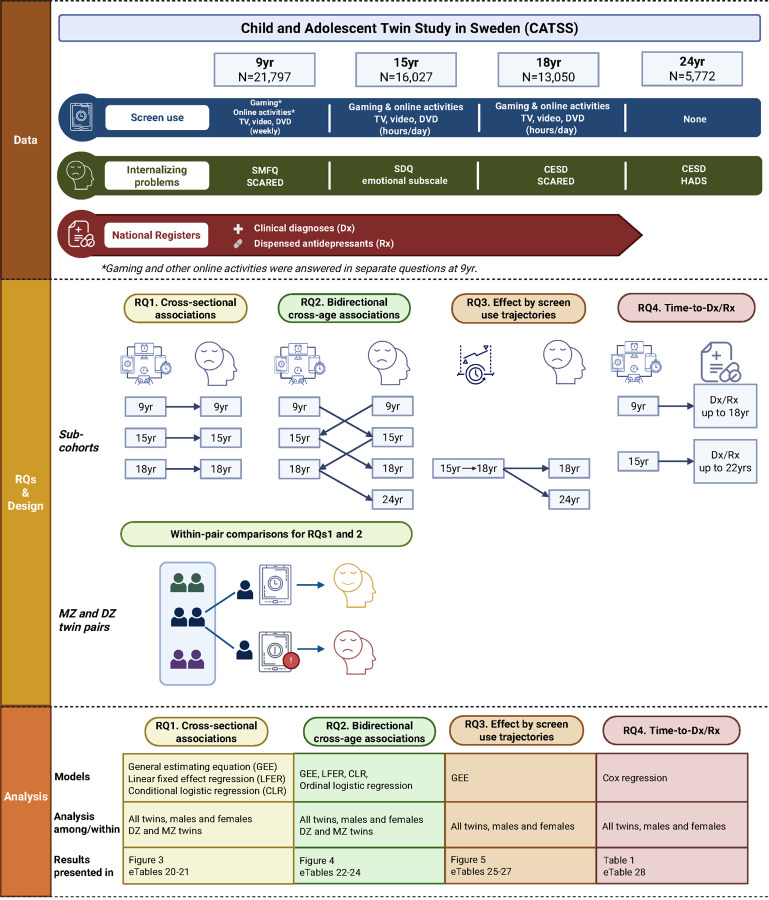
Study overview.

This study aims to clarify the link between recreational screen use and internalizing problems using a large Swedish longitudinal twin cohort: the Child and Adolescent Twin Study in Sweden (CATSS), consisting of approximately 22 000 twins^22,23^. Survey data of recreational screen use and internalizing problems were collected from preadolescence to early adulthood (age 9, 15, 18, and 24), enabling the examination of age- and sex-specific associations across critical developmental stages. Additionally, CATSS data were linked to clinically ascertained diagnoses and pharmacy-dispensed medication records from national registers, allowing for examinations of both parent- or self-reported symptoms and clinical outcomes. Specifically, we address the following research questions (RQ) (Figure 1):

1. Is recreational screen use associated with internalizing symptoms in childhood, mid and late adolescence, and to what extent are these cross-sectional associations attributable to shared genetic and familial environment factors?
2. Does earlier screen use influence later internalizing symptoms across ages, and conversely, do earlier internalizing symptoms influence subsequent screen use? Again, to what extent are these cross-age associations attributable to shared familial factors?
3. Which screen use trajectory (*e.g*., consistent, increasing, or decreasing over time) during mid to late adolescence are most strongly associated with subsequent internalizing symptoms?
4. How does childhood and adolescent screen use affect subsequent risks of clinical depression and anxiety disorders?

## Methods

### Study population

We used data from the Child and Adolescent Twin Study in Sweden (CATSS), an ongoing cohort study with an overall response rate of 60%^22,23^, including information reported by parents and twins through structured telephone interviews and questionnaires on social environment, behavioral traits, somatic and mental health. All twins born in Sweden from July 1992 are invited to participate when they reached 9 years of age (referred to as CATSS-9; for the first three years after the study launch, data were instead collected from twins at 12 years), and subsequently at 15 (CATSS-15), 18 (CATSS-18), and 24 (CATSS-24) years of age. Data collection began in 2004 and by the time of analyses, we included those who had responded to CATSS-9 by May 2020 and CATSS-15, −18, −24 by July 2023. Two subsets of participants from CATSS-9 and −15 who completed the interview/questionnaire before the end of 2015 were also linked to the national registers via unique personal identification numbers^23,24^, which allowed us to retrieve clinical diagnoses, dispensed medications, and mortality data (until the end of 2016) with up to 9 years of follow-up. Further details on the number of participants invited and retained for analysis at each data collection point are presented in **eFigure 1**.

Informed consents were originally provided by the parents, and the twins were repeatedly asked whether they wanted to continue being part of, or to withdraw from the study. The CATSS-study was approved by the Swedish ethical review authority (Dnr 02–289, 03–672, 2010/597–31/1, and 2010/322–31/2, 2010/1114–32, 2011/828–32, 2016/2135–31). We followed the STROBE Reporting Guidelines^25^.

### Measures

#### Recreational screen use

We collected data on twins’ screen use at CATSS-9 (parental report), CATSS-15 (self-report), and CATSS-18 (self-report). Throughout this study, we used ‘screen use’ broadly to encompass different measurements: in CATSS-9, it reflects the frequency of screen-based activities, and in CATSS-15 and CATSS-18, it reflects the time spent on various screen-based activities.

In CATSS-9, parents of the twins completed questions on frequency (‘never’, ’sometime a month’, ‘1–2 times/week’, ‘3–6 times/week’, ‘daily’) of their child engaging in different screen-based activities: 1) online surfing, chatting, and downloading music, 2) watching TV, videos, or DVDs, and 3) playing games (see **eTable 1** for detailed questions being asked at each time point). For gaming, we combined the answers from two separate questions—one on computer gaming and one on video gaming—by selecting the highest reported frequency from either question^26^. We categorized each item into ≤2 times/week (as *reference*), 3–6 times/week, and daily.

In CATSS-15 and CATSS-18, twins self-reported the number of hours (‘never’, ‘<1h/day’, ‘1–2h/day’, ‘2–4h/day’, ‘4–6h/day’, ‘>6h/day’) they spent on each type of activity: 1) watching TV (also online), video, and DVD, 2) playing computer games, video games, chat, blog or browsing the internet, on weekdays and weekends respectively. We categorized each item into ≤2 hours/day, 2–4 hours/day, 4–6 hours/day, and > 6 hours/day, by collapsing the three lowest response options. When analyzed as categorical variable, the most “restrictive use” of ≤2 hours/day was considered as *reference* (except for RQ3 analyses where ≤2 hours/day at two timepoints was considered as *reference*). We termed >6 hours/day as “heavy use”. To reflect differences in the nature of screen activities, we labelled TV/video/DVD watching as “*passive* screen activities” and gaming/chatting/blogging/browsing/online surfing as “*interactive* screen activities”, as they have been previously used in the literature to distinguish the traditional media versus new digital media-based activities with different levels of user interaction and engagement^27^.

Additionally, we used the average values of the answers in each question as continuous measures of screen use (*i.e*., 1.5, 4.5, and 7 times/week in CATSS-9; 1, 3, 5, 7 hours/day in CATSS-15 and −18) to explore the potential linear effect of screen use and enhance the interpretability of result as one time per week increase or one hour per day increase (**eTable 1**).

#### Internalizing symptoms and disorders

Questionnaires measuring internalizing problems, *i.e*., depression- and anxiety-related symptoms were collected at all ages, although with different instruments (see **eTable 2** for detailed information on number of items, coding, score ranges, and data collection periods for each instrument). Briefly, in CATSS-9, parents reported their children’s current depressive and anxiety symptoms from two validated screening instruments: the short mood and feeling questionnaire^28^ (SMFQ) and the screen for child anxiety related emotional disorders^29^ (SCARED). In CATSS-15, we used self-reported current broad emotional problems from the Strengths and Difficulties Questionnaire^30^(SDQ, subscale of emotional symptoms). In CATSS-18, twins completed questionnaires of the Center for Epidemiologic Studies Depression scale^31^(CESD) and the SCARED^29^. In CATSS-24, twins completed questionnaires of the CESD-revised^32^ and the hospital anxiety and depression scale (HADS)^33^.

To assess the clinical outcomes of internalizing conditions (depressive disorders and anxiety disorders), we retrieved diagnostic and medication dispense records from the National Patient Register and the Prescribed Drug Register (see detailed information in **eMethods** and **eTable 3** for the register description as well as the diagnostic and drug codes). Specifically, we defined the clinical outcomes for CATSS-9 and −15 participants according to 1) the date of hospital discharge or outpatient specialist visit with first recorded diagnosis of depressive disorders or anxiety disorders; and/or 2) the dispensing date of the first package of antidepressants (as a proxy for general practitioner visit). We also extracted the dispensing date of the second antidepressant as a secondary outcome for sensitivity analyses, as single antidepressant dispensing may indicate mild or no persistent symptoms, or off-label use due to other indications such as pain or insomnia^34^.

#### Covariates

We obtained survey-based information on twins’ birth year, sex, zygosity, birth country and highest attained education of each parent, and civil status of the responding parent at the time of survey. Additional parent-reported answer to the Life Stressor Checklist-Revised in CATSS-9 was used to capture any of six adverse childhood experiences^17^ (covariate details described in **eMethods**).

### Statistical Analysis

We first present descriptive statistics of participants’ characteristics based on each screen use measure at each time point. Polychoric and Spearman correlations were used to estimate within-twin-pair correlation/concordance for screen use and symptom measures within all, MZ, same-sex DZ and opposite-sex DZ twins.

All symptom measures were standardized to z-scores before association analyses to facilitate effect comparisons across different scales or time points.

To examine associations between screen use with depressive and anxiety symptoms, we used generalized estimation equations (GEE) with clustered robust standard errors to account for twin relatedness. We analyzed three types of associations: (1) cross-sectional associations at single time points (*e.g*., screen time exposures and outcomes at age 9/15/18); (2) cross-age associations (*i.e*., exposures at age 9/15/18 and outcomes at age 15/18/24); and (3) associations between screen use trajectories during adolescence (age 15 to 18) and outcomes at 18 or 24 (Figure 1). To assess screen use trajectories, we used the same measures in CATSS-15 and −18 (age 9 not included due to different measures) to capture all possible combinations of answers (4×4) from the same individual. These combinations were further categorized into six mutually exclusive screen use trajectories across age to facilitate interpretations: constantly restrictive use according to international guidelines^3–7^ (*i.e*., ≤2 hours/day), increased use from ≤2 hours/day, decreased use to ≤2 hours/day, constantly moderate-to-heavy use (*i.e*., >2 hours/day), increased use from >2 hours/day, and decreased use to >2 hours/day.

We considered twins’ birth year and sex, and parental country of birth, highest education, and civil status (when available) as confounders (see **eFigure 2** for a directed acyclic graph motivate covariate selection) and adjusted for them in all models. In addition, we accounted for the pre-existing differences in internalizing symptoms to avoid circular effects in the reverse direction when estimating the effects on later symptoms between different screen exposure groups. Specifically, we controlled for depression/anxiety symptoms reported at an earlier time point in all analyses (*e.g*., the cross-sectional association between screen time and depression/anxiety symptoms at age 15 was further adjusted for the symptoms at age 9), except that we did not have prior symptoms before age 9 – instead, we adjusted for a key risk factor of childhood adverse experiences^35^ reported in CATSS-9. However, the screen use variables at earlier time points could not be adjusted for when modelling mid and late adolescent data due to multicollinearity issues. We estimated the effects of each screen use activity, on weekdays and weekends, in separate models. As results for weekday and weekend use at ages 15 and 18 were similar, we presented weekend estimates in the main text and reported weekday estimates in the **Supplementary Materials.**

Furthermore, we conducted the cross-sectional and cross-age analyses described above using the co-twin control design to account for shared etiological factors. Specifically, within DZ twin pair associations are automatically adjusted for all confounding by shared in-utero and childhood environmental factors and half of their genetic liabilities, while the associations within discordant MZ pairs are additionally controlled for all genetic factors^21^. We conducted analyses within discordant twin pairs using fixed-effect linear regression models, further adjusting for sex (if opposite-sex twin pair), and childhood adverse experiences or prior depression/anxiety symptoms.

To investigate sex-specific associations, we stratified analyses by sex. We also tested whether there were significant sex differences through interaction terms in the adjusted models and presented the interaction p-values.

To examine reverse associations between screen use and depression/anxiety symptoms, we tested the other direction in cross-age analyses (*i.e*., depression/anxiety symptoms at 9/15 and screen use at 15/18). We applied ordinal logistic regression models with sandwich estimators to account for twin clustering, and the models were adjusted for birth year, sex, parental country of birth, education, civil status, and screen use at prior time point.

Lastly, to assess the association of screen use with clinical conditions of depressive disorders and anxiety disorders, we conducted survival analyses using Cox regression models in CATSS-9 and −15. We excluded prevalent cases before the start of follow-up. The censoring and modelling details are provided in **eMethods**. SAS 9.4 was used for data management and R-4.3.2 (R Foundation for Statistical Computing) was used for all analyses. Data were analyzed during June-December 2024.

## Results

This prospective study included 21 797 twins (response rate 68.5%, 49.9% females) whose parents initially participated in the CATSS-9 telephone interview. Among these, 16 027 (73% of the CATSS-9 twins), 13 050 (60%), and 5 772 (27%) twins completed CATSS-15, −18 and −24 questionnaires, respectively (**eFigure 1**). At age 9, the majority (66%) watched TV, video, and DVD daily, and a higher proportion of boys (38%) played games daily compared to girls (23%). Similarly, at ages 15 and 18, more boys than girls engaged heavily in interactive screen activities (*i.e*., gaming, chatting, and browsing the internet), particularly during weekends (*e.g*., in CATSS-15, 23% of boys versus 12% of girls used >6 hours/day, **eFigure 3**). In contrast, patterns of passive screen activities (*i.e*., watching TV, video, and DVDs) were similar between boys and girls.

Comparing participant’s characteristics, heavy use of either passive or interactive screen activities (*i.e*., >6 hours/day at age 15/18) was less common among those whose parents had university education. Compared to those who reported restrictive use (*i.e*., ≤2 hours/day), participants who reported heavy screen use also reported higher internalizing symptom scores at both prior and current ages (**eTables 4–18**).

**Figure 2:**
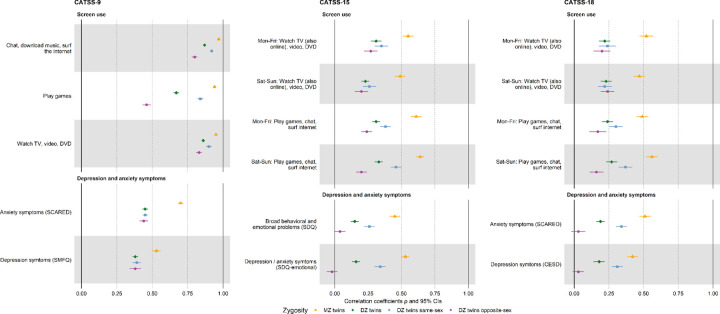
Within-twin-pair correlations of recreational screen use and depression/anxiety symptoms. Within-twin-pair correlations on recreational screen use and depression/anxiety symptoms based on parent-reported CATSS-9 (left panel), and self-reported CATSS −15 (middle panel) as well as CATSS-18 (right panel) data. Polychoric or Spearman’s correlation coefficients and 95% CIs are presented for MZ (yellow) and DZ twins (green) overall, as well as same-(blue) and opposite-sex DZ twins (purple).

Within-pair correlations for parent-reported screen use at age 9 (correlation coefficients ρ ranging 0.94–0.97 for MZ and 0.46–0.92 for DZ twins) were stronger than those for self-reported at age 15 (ρ_(MZ)_ 0.49–0.61 and ρ_(DZ)_ 0.20–0.46) or 18 (ρ_(MZ)_ 0.47–0.56 and ρ_(DZ)_ 0.16–0.37), and similarly for depressive and anxiety symptoms. Across all ages and measures, correlations were stronger within MZ than within DZ twins, suggesting genetic influences. Sex differences were observed in twin correlations, particularly for screen activities involving gaming at age 9 (ρ_(DZ)_ 0.84 versus 0.46 among same-sex versus opposite-sex DZ twins), and depressive/anxiety symptoms at age 15 and 18 (Figure 2; **eTable 19**).

### Cross-sectional associations between recreational screen use and internalizing problems

We first tested associations between screen use and internalizing symptom scores at ages 9, 15, and 18, both in the full twin cohort and within discordant DZ and MZ twin pairs.

At age 9, we observed associations with depressive and anxiety symptoms across different types of screen use, when comparing the most frequent use (daily) to the restrictive use (≤2 times/week) (**eTable 20**). No significant sex differences were observed at this age (**eTable 21**). Furthermore, the high within-twin correlations of parent-reported screen use (Figure 2) limited the number of discordant twin pairs, yielding mostly non-significant estimates within DZ and MZ twins (**eTable 20**).

**Figure 3:**
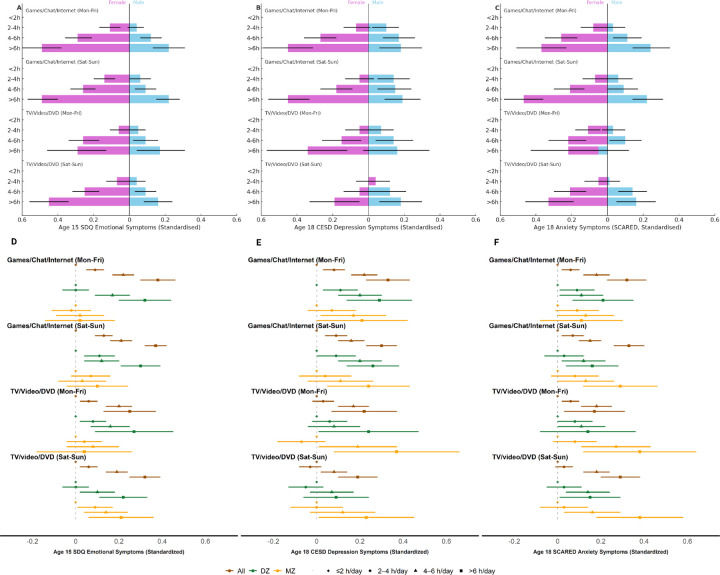
Cross sectional associations of recreational screen use with depressive and/or anxiety symptoms at ages 15 and 18. Cross-sectional associations of recreational screen use (upper panel A-C on estimates separately by sex and lower panel D-F on estimates among all participants and within discordant MZ and DZ twin pairs) with depression/anxiety symptoms based on self-reported data in CATSS-15 (left column for depression/anxiety) as well as CATSS-18 (middle and right columns for depression and anxiety respectively). Effect estimates for all twins (brown), female-specific (pink), and male-specific (blue), *i.e*., standardized beta coefficients and 95% CIs by screen use frequencies are presented after fitting generalized estimating equation, with adjustment for birth year, sex (if not stratified), parental birth countries, highest education, and civil status, as well as twins’ prior depressive/anxiety symptoms. Within-pair-comparisons are also presented in standardized beta coefficients and 95% CIs after fitting the fixed effect models within DZ (green), and MZ (yellow) twin pairs separately and adjusting for sex (if not same-sex pairs) and prior depressive/anxiety symptoms.

At ages 15 and 18, the association between screen time and internalizing problems in all twins demonstrated a dose-response pattern, with longer screen time per day corresponding to higher symptom scores (**eTable 20**). Notable sex differences were observed in these associations, particularly for interactive screen activities. The effect estimates were much larger in girls than boys: compared to the most restrictive use (≤2 hours/day), heavy use (>6 hours/day) was associated with standardized scores ranging between 0.37–0.49 in girls and 0.18–0.24 in boys (Figure 3A–C; **eTable 21**).

In discordant twin comparisons at ages 15 and 18 (Figure 3D–F), we found slightly attenuated effects within DZ pairs and further attenuation within MZ pairs [*e.g*., heavy use of interactive screen activities during weekends was associated with standardized β (95% CIs) on age 18 CESD scores: 0.30 (0.23–0.37) for all, 0.26 (0.14–0.38) within DZ and 0.24 (0.05, 0.43) within MZ twins] (**eTable 20**). Nevertheless, most associations remained significant within discordant twin pairs, where shared genetic confounding and familial environment factors were implicitly accounted for (Figure 3D–F; **eTable 21**). When comparing within DZ and MZ same-sex pairs, the estimates were often stronger among girls than boys, although such sex difference was less clear than observed in analyses with all twins (**eTable 21**).

### Bidirectional cross-age associations between recreational screen use and internalizing problems

Next, we conducted analyses on cross-age associations to better understand directionality. Among twins who participated in at least two surveys at different ages, we explored the link in both directions: from earlier screen use to later internalizing symptoms, and from earlier symptoms to later screen use. Similarly, we performed discordant twin comparisons to further account for shared genetic and family environmental factors, though here we analyzed DZ and MZ twins together due to the reduced sample sizes when requiring complete data at different ages.

**Figure 4:**
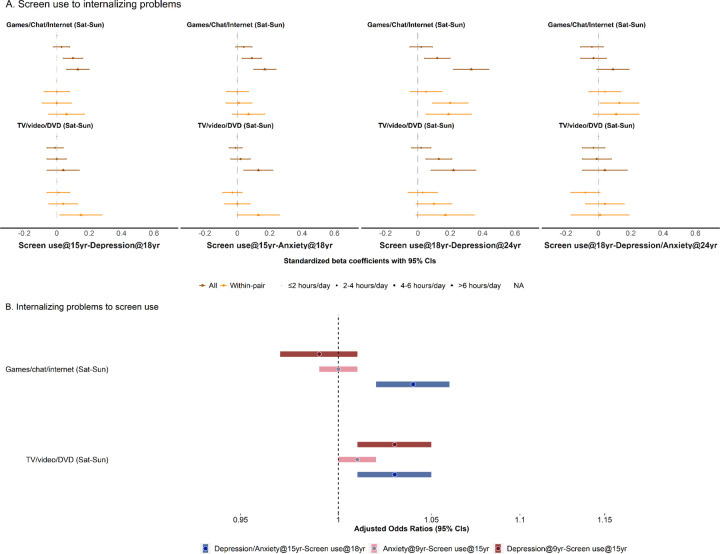
Bidirectional cross-age associations of recreational screen use during weekends with symptoms of depression and anxiety. A. Cross-age associations of recreational screen use at age 15/18 with internalizing problems at age 18/24. Standardized beta coefficients for depression and/or anxiety symptom (diamond for ≤2 hours/day, circle for 2–4 hours/day, triangle for 4–6 hours/day, and square for >6 hours/day in brown) are presented after fitting generalized estimating equation among all eligible twin participants, with adjustment for birth year, sex, parental birth countries, education, and civil status, as well as twins’ prior depressive/anxiety symptoms. Within-pair estimates are (similar signs but in orange) also presented after fitting fixed effect models within discordant twin pairs and adjusting for sex and prior depressive/anxiety symptoms. B. Reverse associations between internalizing problems at age 9/15 and recreational screen use at age 15/18. Odds ratios (OR) and 95% CIs represented the odds of shifting towards heavier screen use by more depressive/anxiety symptoms (*i.e*. 1 SD increase on the standardized depression/anxiety scores). Estimates are presented after fitting ordinal logistic regression, adjusting for birth year, sex, parental birth countries, highest education, and civil status, as well as twins’ prior screen use frequencies.

First, screen use at age 9 was not significantly associated with internalizing symptoms at 15, whereas screen use at ages 15 and 18 showed consistent associations with symptoms at ages 18 and 24, respectively (Figure 4A; **eTable 22**). Particularly, heavy use of interactive screen activities at age 15 was strongly associated with elevated depressive or anxiety symptoms at age 18 (mean standardized symptom scores were 0.15–0.30 unit higher compared to those with restrictive use). Similarly, heavy use of interactive screen activities at age 18 was associated with more depressive symptoms (0.29–0.35 higher on standardized CESD scores) reported at age 24. In comparison, heavy use of passive screen activities at ages 15 and 18 showed weaker associations with subsequent symptoms. While most associations remained significant in sex-stratified analyses, we did not observe a pattern of consistently stronger association among girls than boys as shown in the cross-sectional analyses (**eTable 23**).

Furthermore, the cross-age effects within discordant twin pairs were attenuated compared to the estimates from the full cohort. Nonetheless, heavy use of interactive screen activities at age 18 remained significantly associated with depressive symptoms at age 24 (0.19 higher on standardized CESD scores compared to the restrictive use; Figure 4A; **eTable 22**).

For reverse associations between experiencing depressive and anxiety symptoms at ages 9 and 15 and subsequent screen use at ages 15 and 18 (Figure 4B; **eTable 24**), we observed weak but positive association (OR=1.03 [1.01–1.05]) for age-9 depressive symptoms increasing the age-15 screen use of TV, video, and DVD, as well as age-15 depressive/anxiety symptoms increasing the age-18 screen use in general (ORs between 1.03–1.04). No clear sex difference was observed (**eTable 24**).

### Changes of recreational screen use and internalizing symptoms

With consistent measures of screen use at ages 15 and 18, we investigated the trajectories of screen use to better understand the dynamics of screen exposure over this developmental period and their associations with internalizing symptoms at ages 18 and 24.

**Figure 5:**
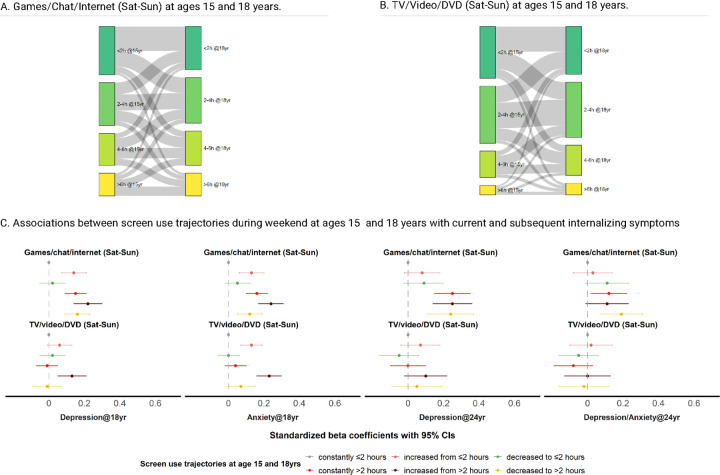
Recreational screen use trajectories during weekends at ages 15 and 18 with current and subsequent internalizing symptoms at ages 18 and 24. A-B. Two Sankey diagrams illustrate the patterns of weekend screen use between age 15 and 18 (darker green for ≤2 hours/day, green for 2–4 hours/day, lighter green for 4–6 hours/day, yellow for >6 hours/day) involving gaming, chatting, browsing internet, and watching TV (also online), video, and DVD. Grey lines depict the physical flows of participants associated with each screen use categories at two time points. C. Associations between screen use patterns during weekends (grey for constantly ≤2 hours/day, pink for increased from ≤2 hours/day, green for decreased to ≤2 hours/day, red for constantly >2 hours/day, maroon for increased from >2 hours/day, and yellow for decreased to >2 hours/day) with symptoms of depression and/or anxiety. Standardized beta coefficients for depressive and/or anxiety symptoms with 95% CIs are presented after fitting generalized estimating equations, and adjusting for birth year, sex, parental birth countries, highest education, and civil status, as well as twins’ prior depressive/anxiety symptoms.

The frequency of each screen use category remained relatively stable between ages 15 and 18, with the highest proportion of participants reporting screen time within the same levels. However, frequent transitions were also observed with both increasing and decreasing screen time from age 15 to 18 (Figure 5A–B; **eFigure 4**).

We then explored how these screen use trajectories from age 15 to 18 were associated with current and subsequent internalizing symptoms at ages 18 and 24. In light of international recommendations^3,5–7^ on screen time for adolescents, here we considered individuals who remained within the ≤2 hours per day limit at both 15 and 18 as the reference group (**eFigure 5; eTable 25**). Adolescents who spent >2 hours on recreational screen time at both ages (*i.e*., constantly >2, increased from >2, and decreased to >2 hours), particularly on interactive activities, scored higher on subsequent internalizing symptoms than the reference group. For example, effects on depressive symptoms ranged from 0.14 to 0.39 higher on the standardized CESD scores. Interestingly, those whose interactive screen time increased from ≤2 at age 15 to >2 hours at age 18 (*i.e*., the newly exceeding the recommended limit group) showed modest but generally significant increases in internalizing symptoms at both 18 and 24 years (*e.g*., 0.03–0.15 higher on standardized CESD). In contrast, adolescents who decreased to ≤2 hours (*i.e*., reduced screen use to the recommended limit) showed mostly null or weak associations (Figure 5C; **eTable 26**). Furthermore, we did not observe significant sex differences for screen use trajectories (**eTable 27**).

### Screen use and subsequent risks of clinically ascertained depressive disorders and anxiety disorders

We relied on self-reported internalizing problems in earlier analyses. To better understand the associations with clinical outcomes, we conducted survival analyses with time-to-event outcomes of clinically ascertained depressive disorders and anxiety disorders (see *Methods* and *eMethods*).

Among 15,470 CATSS-9 participants, we identified 4.6% with any of the clinical outcomes during the follow-up period from the completion of the age 9 survey up to age 18. Similarly, among 9,373 CATSS-15 participants, we identified 10.4% with any of the clinical outcomes during the follow-up up to age 22 (**eFigure 6**). The unadjusted incidence rates and cumulative incidences of most outcomes were highest among the heavy use (daily or >6 hours/day) groups (**eTable28**; **eFigures 7–13**). Due to the relatively low number of events particularly in the heavy use group, we did not perform discordant twin comparisons in these analyses.

We found that playing games daily at age 9 (parental-report) compared to restricted use (<2 times/week was significantly associated with higher risk of clinically ascertained depressive or anxiety disorders (adjusted hazard ratio, aHR in both sexes: 1.23, 95% CI: 1.00–1.52; aHR in boys: 1.37, 1.00–1.87, **eTable 28**). In comparison, daily screen activities of chat, downloading music, surfing internet, or watching TV, video, and DVD were not significantly associated with these clinical outcomes.

At age 15, heavy interactive screen use during weekdays was associated with 86% increased risk when compared to those with restricted use (aHR =1.86, 95% CI: 1.41–2.45; heavy use during weekends: aHR = 1.37, 95% CI: 1.09–1.72). Heavy use of passive screen activities at age 15 during weekdays was also associated with a similar risk. Most of these associations were found in boys and girls separately; however, with wide confidence intervals, we cannot test sex difference in these estimates (Table 1; **eTable 28**).

## Discussion

In this large population-based twin cohort spanning pre-adolescence to young adulthood, we found that recreational screen use, particularly during adolescence, was associated with increased internalizing symptoms and clinically diagnosed depressive and anxiety disorders. Cross-sectional associations were strongest at ages 15 and 18 and more pronounced among girls, even after adjusting for prior symptoms and shared genetic and familial environmental factors (RQ1). Cross-age analyses revealed a bidirectional association between screen use and internalizing symptoms around mid-adolescence to young adulthood (RQ2). Consistently high or increasing screen use, compared with stable restrictive use, between ages 15 and 18 was associated with elevated symptoms at later ages; in contrast, the association did not persist when screen use decreased to a restrictive level (RQ3). Finally, heavy use, especially of interactive screen activities, was linked to higher risks of clinical conditions of depressive and anxiety disorders (diagnosis or antidepressant dispensing), further underscoring its potential clinical and public health significance (RQ4).

Behavioral genetics research has consistently shown that behavioral traits such as mental health symptoms^36^ and screen-related behaviors, including gaming^26^, TV watching^37^, computer and online media use^37,38^, are influenced by genetic factors^39^. Our results of higher MZ correlations of screen use and internalizing symptoms than DZ correlations are in line with this literature. As such, individual genetic differences may represent an important source of unmeasured confounding in the association between screen use and youth mental health; yet, these have rarely been accounted for in previous literature. Two recent studies using genetically-informed design have demonstrated that screen and media use behaviors are moderately heritable (heritability estimates of 20–49%)^38,40^ and that genetic confounding explains a substantial portion of the associations with internalizing problems^40,41^. In parallel, epidemiological studies have considered family environment, such as sociodemographic and maternal characteristics, as confounders in these associations^42–46^. However, other familial influences, such as parenting practices^47^, are often difficult to measure or omitted altogether, complicating the interpretations of observational findings^16^. Consistent with these concerns, we observed attenuated associations in discordant DZ and MZ twin comparisons, supporting a role of shared genetic and environmental liability. Nonetheless, the observed associations in our cohort largely persisted within twin pairs cross-sectionally in mid-to-late adolescence, and cross-age from late adolescence to early adulthood, particularly for the heavy interactive screen use. These findings suggest that increased screen use may independently contribute to internalizing problems beyond shared familial factors.

Consistent with previous longitudinal studies^43,44,48^, our cross-age analyses confirmed that heavy use during adolescence, particularly on interactive screen activities, was associated with elevated internalizing problems at later ages. In our data, childhood screen use at age 9 was not significantly associated with subsequent symptoms during adolescence, which may reflect developmental differences in how children engage with screens (more passive and/or parent-regulated activities at age 9 compared to adolescence), or potential discrepancies between parent- and self-reported measures^12^.

Conversely, we observed modest reverse associations: individuals with higher internalizing symptoms at ages 9 and 15 showed small increases of screen use at the next follow-up (ORs 1.03–1.04). Despite the modest effect sizes (particularly when compared to the effect sizes in the other direction), these results support increased attention to screen use behaviors in adolescents already experiencing symptoms, particularly in clinical subgroups for whom research remains critically lacking^11^. Recent reports showed that, beyond simply using screens more, adolescence with internalizing conditions reported more negative social media experiences, such as greater scrolling-induced insecurity and social comparison, as well as stronger mood impact, compared with their peers with no mental health conditions^49–51^. Together, these findings, along with previous literature^52^, emphasize the specific need of focusing on clinically vulnerable subgroups and developing targeted interventions (for example, coping strategies for negative feedback or social comparison) to prevent severe outcomes, including the rising incidence of self-harm among youth^14,53^.

Causal evidence is essential to ensure that public health guidelines and interventions target true modifiable risk factors rather than mere correlates^54^. However, RCTs that evaluate adolescent screen use over an extended period remain scarce, leaving a gap in causal understanding and thus limiting the ability to confidently evaluate the effectiveness of current guidelines. In the absence of such trials, the field has called for robust causal inference methods applied to observational data, including natural experiments^10,55^. In this study, we leveraged a co-twin control design and triangulated evidence across multiple analytic approaches (cross-sectional, cross-age, and survival analyses) to strengthen causal inference between screen use and internalizing problems. Further, we assessed screen use trajectories between ages 15–18 in relation to international recommendations, which mostly advise limiting recreational screen time to ≤2 hours per day (or 2–3 hours in the Swedish guideline) for this age range^3–7^. Compared with adolescents who remained below this threshold at both ages, those whose screen time were increased beyond it reported significantly higher internalizing symptoms at age 18, whereas those who reduced to the recommended level showed no significant symptom elevation. Our findings are in line with results from a recent target trial emulation—among several modifiable factors (including screen time, sleep, physical activity, and social connectedness), screen time was most consistent with having a causal role: more stringent threshold (0–1 h/day) associated with lower internalizing symptoms, while more lenient threshold (3–4 h/day) increased symptoms^56^. These findings together support the hypothesis that adhering to public health guidelines may contribute to the mitigation of internalizing symptoms in adolescence. Nevertheless, further research using diverse causal inference strategies is needed to corroborate these associations and inform evidence-based interventions.

Researchers have also debated whether the effects of screen time are “too small to warrant policy change”^57–59^. Consistent with previous literature^13^, we found relatively small effect sizes associated with per-hour increase, while the impact on internalizing symptoms was most pronounced among those in the heavy use group and among adolescent girls. However, symptom elevations do not necessarily translate into clinical conditions^15^, raising the question of whether these effects are clinically meaningful. To address this, we linked screen use at ages 9 and 15 with clinically ascertained outcomes over longitudinal follow-up. We found that daily gaming at age 9 was associated with a 23% higher risk of clinically ascertained depressive or anxiety disorders by age 18, and that heavy interactive screen use at age 15 conferred an 86% increased risk of these disorders by early adulthood. Passive screen use at age 15 also increased the risk, although to a lesser extent. Intriguingly, we did not observe stronger associations among girls than boys, potentially due to inadequate statistical power or differential patterns in help-seeking or clinical detection. Assuming a similar degree of effect attenuation due to shared familial factors (as observed in symptom-based co-twin analyses), these results suggest that high screen engagement during adolescence have clinically meaningful implications for the onset of internalizing disorders. This extends prior symptom-based evidence to clinical outcomes, reinforcing the clinical relevance of reducing excessive screen use during this key developmental period.

This study has several notable strengths, including the use of a large, population-based twin sample with longitudinal assessments, and the triangulation of causal evidence using multiple epidemiological designs, such as cross-sectional, co-twin control, cross-age/trajectory, and survival analyses. We, however, acknowledge the following limitations. First, the CATSS data collection started in 2004, *i.e*., before the widespread adoption of smartphones and social media, and we did not examine secular trends, which might have influenced the associations over time. Second, our crude measures of screen time do not capture the complexity of digital media use^60,61^. The rapidly changing digital landscape affects screen use patterns, and motivations behind screen use (*e.g*., gaming for social connection vs. scrolling for emotional escape) are likely central to understanding its link with mental health^62^. Third, general underestimation of screen use, particularly the underreporting of smartphone usage^63,64^, may lead to an underestimation of the true associations. However, we found that approximately 24% of participants on weekdays and 40% on weekends reported >4 hours/day of interactive screen activities at age 15—compared with 14% and 27% respectively who were heavily engaged with passive screen activities. These prevalences are consistent with two Swedish studies^65,66^ and are broadly comparable to findings among adolescents in the U.S. and U.K.^42,43,67^. Fourth, residual confounding from non-shared factors may have influenced the findings. For example, physical activity, peer relationship, and social environments during adolescence could displace screen use and may vary within twin pairs. Such activities could partially modify the observed associations, if not acting as mediators in the causal pathways. Similarly, substitution between different types of screen activities may also be a concern. Here we analyzed the interactive and passive screen activities separately aiming to directly compare the effects across the two domains. Future studies employing advanced modelling are needed to address the complex relationship^51^. Lastly, as in other observational data, twin data are powerful but not immune to methodological limitations^68,69^. Measurement errors within twin pairs on screen use may bias estimates and the declining response rates (~70% to ~50%) over time may introduce selection bias.

## Conclusion

Taken together, this study used methodological triangulation which allowed for a rigorous examination of the potential causal link between recreational screen use and internalizing problems, and findings showed bidirectional associations from preadolescence to young adulthood. Future research should explore the differential effects of engaging with various types of screen and non-screen activities and how individual vulnerabilities may interact with sex and developmental stage. Our findings emphasize heavy recreational screen use as a growing public health and clinical concern, and highlight the need for clearer, evidence-based guidance to maintain safer recreational screen use in children and adolescents.

## Supplementary Material

Supplementary Files

This is a list of supplementary files associated with this preprint. Click to download.

- STRscreentimedepanxsupplementarymaterials20250801.docx
- Supplementarytables20250801.xlsx

## Figures and Tables

**Table 1.** Hazard Ratios (HR) and 95% Confidence Intervals for depressive and anxiety disorders by recreational screen use at age 15 among CATSS-15 participants (n=15 470).

Exposures	Exposure levels	Nr of events	All^†^	Female^†^	Male^†^
Games/Chat/Internet (Mon-Fri)	≤2 hours/day	432	Ref	Ref	Ref
	2–4 hours/day	279	1.16(0.98, 1.38)	1.18(0.96, 3.99)	1.14(0.82, 4.36)
	4–6 hours/day	170	**1.27(1.04, 1.57)**	**1.31(1.03, 4.74)**	1.21(0.83, 4.87)
	>6 hours/day	83	**1.86(1.41, 2.45)**	**1.63(1.14, 7.28)**	**2.21(1.42, 14.22)**
Games/Chat/Internet (Sat-Sun)	≤2 hours/day	323	Ref	Ref	Ref
	2–4 hours/day	263	1.02(0.84, 1.22)	0.99(0.80, 3.33)	1.11(0.73, 4.62)
	4–6 hours/day	213	1.22(0.99, 1.48)	1.20(0.95, 4.20)	1.30(0.86, 5.55)
	>6 hours/day	165	**1.37(1.09, 1.72)**	**1.36(1.02, 5.21)**	1.45(0.95, 6.48)
TV/Video/DVD (Mon-Fri)	≤2 hours/day	577	Ref	Ref	Ref
	2–4 hours/day	263	0.89(0.76, 1.06)	0.88(0.73, 2.93)	0.92(0.67, 3.43)
	4–6 hours/day	97	0.88(0.67, 1.15)	0.93(0.68, 3.48)	0.75(0.43, 3.70)
	>6 hours/day	27	**1.57(1.02, 2.41)**	1.34(0.79, 6.51)	2.30(1.10, 20.8)
TV/Video/DVD (Sat-Sun)	≤2 hours/day	374	Ref	Ref	Ref
	2–4 hours/day	370	0.88(0.75, 1.04)	0.90(0.74, 2.99)	0.83(0.61, 3.13)
	4–6 hours/day	151	0.78(0.63, 0.97)	0.77(0.59, 2.80)	0.79(0.53, 3.30)
	>6 hours/day	69	1.09(0.81, 1.47)	1.14(0.79, 4.50)	0.98(0.57, 4.59)

† Models adjusted for birth year, sex, parental birth country, highest education and civil status, as well as depression/anxiety symptoms at 9 year, and sex-specific estimates were presented based on stratified analyses.

## Data Availability

The data used in this study are derived from the Swedish Twin Registry (STR), which is governed by strict data protection laws in Sweden. Due to the legal and ethical restrictions, the data cannot be made publicly available. Access to the STR data requires a formal application to the STR Steering Committee and approval from the Steering Committee and the Swedish ethical review authority. The authors obtained access to de-identified data under these conditions and are not permitted to share the dataset. Researchers interested in accessing the STR data for replication or further research may submit a project proposal and an ethic application as outlined by the STR (see https://ki.se/en/research/research-infrastructure-and-environments/core-facilities-for-research/swedish-twin-registry-core-facility).
